# Effect of a Structured Preoperative Educational Video on Maternal Anxiety in Elective Cesarean Section: A Randomized Controlled Trial Using EEG Biomarkers

**DOI:** 10.4274/TJAR.2026.262590

**Published:** 2026-08-28

**Authors:** Ashutosh Kaushal, Tamonud Modak, Anuj Jain, Shikha Jain, L Pfokreni, Ujjwal Gupta

**Affiliations:** 1 All India Institute of Medical Sciences, Department of Anaesthesiology and Critical Care, Bhopal, India; 2 All India Institute of Medical Sciences, Department of Psychiatry, Bhopal, India

**Keywords:** Cesarean section, education of patients, electroencephalography, anxiety, video-audio demonstration

## Abstract

**Objective:**

Preoperative anxiety is common in patients undergoing elective cesarean section and may worsen perioperative outcomes. This study evaluated whether a structured preoperative educational video (SPEV) reduces maternal anxiety, as measured by the Amsterdam Preoperative Anxiety and Information Scale (APAIS) and the quantitative electroencephalography (QEEG)-derived theta-beta ratio (TBR).

**Methods:**

Ninety primigravida women undergoing elective cesarean section with spinal anaesthesia were randomized to a video group (n = 45; SPEV plus routine counseling) or a control group (n = 45; routine counseling only). APAIS was measured at baseline and 30 minutes before surgery. Parietal TBR was recorded at baseline, 30 minutes before surgery, and 1 hour after surgery. Groups were compared using appropriate non-parametric tests, and the correlation between subjective and QEEG-derived measures was assessed.

**Results:**

Baseline characteristics were comparable between groups. At 30 minutes before surgery, APAIS scores were lower in the video group than in the control group [11.0 (9.0-12.0) vs. 14.0 (12.0-16.0); *P* < 0.001). TBR was also lower before surgery (1.20 vs. 1.90; *P* < 0.001) and at 1 hour postoperatively (1.20 vs. 1.80; *P* < 0.001). A positive correlation between APAIS scores and TBR was observed 30 minutes before surgery (*P*=0.48, *P* < 0.001). Intraoperative midazolam requirement was lower in the video group (11.1% vs. 33.3%, *P*=0.021).

**Conclusion:**

A SPEV significantly reduced preoperative anxiety in women undergoing elective cesarean section, as demonstrated by both subjective and QEEG-derived measures, and was associated with reduced intraoperative anxiolytic requirements.

**Main Points**

• A structured preoperative educational video significantly reduced maternal anxiety before an elective cesarean section compared with routine counseling alone.

• Anxiety reduction was demonstrated using both subjective assessment (Amsterdam Preoperative Anxiety and Information Scale) and objective neurophysiological measurement [quantitative electroencephalography-derived parietal theta-beta ratio (TBR)].

• Short, low-cost audiovisual education can be easily integrated into routine obstetric preoperative care using commonly available digital devices.

• Parietal TBR may serve as a reliable objective biomarker for preoperative anxiety.

## Introduction

Preoperative anxiety is a common psychological response among patients undergoing surgical procedures, affecting 60-80% of individuals scheduled for elective operations. In obstetric populations, particularly among women undergoing lower-segment cesarean section (LSCS), anxiety may be even more pronounced due to concerns about maternal safety and fetal well-being, fears related to anaesthesia, and unfamiliarity with the operating room environment. Such anxiety is clinically significant, as it has been associated with increased anaesthetic and analgesic requirements, higher postoperative pain scores, delayed recovery, impaired wound healing, prolonged hospital stay, and reduced patient satisfaction.^1, 2, 3, 4, 5^

Non-pharmacological strategies to reduce perioperative anxiety have gained increasing attention because of their safety, feasibility, and cost-effectiveness. Among these, structured educational interventions, particularly audiovisual tools, have emerged as promising approaches to improve patient understanding and preparedness. Educational videos provide standardized and easily comprehensible information regarding perioperative processes, thereby reducing uncertainty and enhancing patient confidence. In obstetric practice, such interventions may be especially beneficial, improving cooperation during regional anaesthesia and enhancing the overall perioperative experience.^6, 7, 8, 9, 10, 11, 12^

Despite growing evidence supporting the effectiveness of educational interventions, most studies assessing perioperative anxiety rely predominantly on subjective psychometric scales. Objective neurophysiological measures of anxiety remain underexplored in this setting. Quantitative electroencephalography (QEEG) has been increasingly investigated as a tool for assessing emotional and cognitive states, providing real-time insights into cortical activity associated with anxiety. In particular, the theta-beta ratio (TBR) has been proposed as a biomarker reflecting attentional control, emotional regulation, and stress-related cortical dynamics. However, few studies have evaluated, using both validated subjective anxiety scales and QEEG-derived biomarkers, the impact of preoperative educational videos on on women undergoing cesarean section.^13, 14^

Therefore, this randomized controlled trial aimed to evaluate the effect of a structured preoperative educational video (SPEV) on preoperative anxiety among primigravida women undergoing elective LSCS with subarachnoid block (SAB). Anxiety was assessed using the Amsterdam Preoperative Anxiety and Information Scale (APAIS), and the assessment was complemented by quantitative QEEG analysis of the TBR as an objective biomarker of anxiety-related cortical activity. We hypothesized that exposure to a SPEV would significantly reduce both subjective anxiety scores and QEEG-derived TBR compared with routine counseling alone.

The primary objective of the study was to compare preoperative anxiety levels between the intervention and control groups, as measured by the APAIS 30 minutes before surgery. Secondary objectives included the comparison of QEEG-derived TBR at predefined perioperative time points, assessment of the correlation between subjective and objective measures of anxiety, and evaluation of intraoperative midazolam requirements between the two groups.

## Methods

This randomized controlled trial was conducted over a period of six months in the maternity ward and operating theatre complex of the All India Institute of Medical Sciences, Bhopal New Delhi. The study was approved by the Institutional Ethics Committee of the All India Institute of Medical Sciences, Bhopal, New Delhi (approval no: IHEC-LOP/2023/IL0146, date: 02.01.2024). It was then conducted in accordance with CONSORT guidelines after prospective trial registration in the Indian Clinical Trials Registry (CTRI/2024/03/063964). Written informed consent was not required.

Primigravid women scheduled for elective LSCS under SAB were eligible for inclusion. Exclusion criteria included refusal to participate, visual or hearing impairment, requirement for general anaesthesia, history of psychiatric illness or anxiety disorder, use of psychotropic medications, and prior surgical experience. Eligibility was confirmed during pre-anaesthetic evaluation.

The sample size was calculated based on prior studies evaluating preoperative anxiety, assuming a moderate effect size (Cohen’s d = 0.5), with a statistical power of 80% and a two-sided significance level (alpha) of 0.05.^15, 16^ The minimum required sample size was estimated to be 40 participants per group. To account for potential attrition or incomplete data, 45 participants were enrolled in each group, resulting in a total sample size of 90.

Participants were randomized in a 1:1 ratio to the video group (Group V) or the control group (Group C) using a computer-generated random number sequence. Allocation concealment was ensured using sequentially numbered, sealed opaque envelopes prepared by an investigator not involved in recruitment or outcome assessment. The study was single-blind: outcome assessors and QEEG analysts were blinded to group allocation. All participants received routine preoperative counseling and standard peri-anaesthetic care according to institutional protocol.

In Group V, on the day of hospital admission and approximately 24 hours before surgery, the SPEV was shown on a smartphone operated by the investigator after the baseline APAIS assessment. The video was shown in a quiet counseling room in the maternity ward, with audio delivered via headphones or at low volume for clarity and privacy. The perioperative pathway and operating room events were explained in detail during the video presentation. The 6-minute video covered the patient journey, including pre-anaesthetic evaluation, transfer to the operating room, application of standard monitoring, administration of spinal anaesthesia, intraoperative events including fetal delivery, and postoperative recovery and monitoring.

The video was independently reviewed by two consultant anaesthesiologists and one obstetrician for clinical accuracy, completeness, and suitability for patient understanding. Informal pilot testing was conducted with a small group of patients who were not included in the final analysis to assess the clarity, comprehensibility, and duration. Feedback from experts and patients led to minor modifications in language and sequencing to improve patient comprehension. Following the video, participants were encouraged to ask questions, and the investigator provided clarifications.

Group C received standardized verbal counseling delivered by the same investigator. The counseling covered predefined domains that were identical to those in the video content, including pre-anaesthetic evaluation, transfer to the operating room, application of standard monitoring, administration of spinal anaesthesia, intraoperative events including fetal delivery, and postoperative recovery and monitoring. Verbal counseling was delivered according to a predefined script, lasted the same duration as the intervention group (6 minutes), and did not include any additional individualized information. This was done to minimize performance bias related to unequal attention.

Anxiety was assessed using the validated APAIS, which consists of six items scored on a five-point Likert scale.^17^ Assessments were performed at two predefined time points: baseline (upon admission, approximately 24 hours before surgery) and 30 minutes before surgery. APAIS scoring was performed by an investigator blinded to group allocation.

QEEG recordings were obtained at the same time points using an 8-channel system with electrode placement based on the International 10-10 system. An 8-channel EEG system was selected to ensure feasibility in a perioperative clinical setting while maintaining adequate signal quality. Parietal electrodes (P3 and P4) were selected a priori based on evidence suggesting their involvement in anticipatory anxiety and internally directed attention. Recordings were performed with the participant in a seated, relaxed position, with eyes closed. Signals were acquired at a sampling frequency of 250 Hz, band-pass filtered between 0.5 and 45 Hz, and visually inspected to remove artifacts, including eye movements, muscle activity, and electrical noise. Segments with excessive artifacts were excluded from the analysis. Power spectral density was computed using the fast Fourier transform, and band power was calculated for theta (4-8 Hz), alpha (8-13 Hz), and beta (13-30 Hz) frequency bands. The TBR was calculated as the ratio of theta band power to beta band power. Although recordings were obtained from multiple regions, parietal TBR, derived from electrodes P3 and P4, was predefined as the primary QEEG parameter of interest because of its association with internally directed attention and anticipatory anxiety. QEEG analysis was performed by an investigator blinded to group allocation.

All patients underwent cesarean section under spinal anaesthesia, according to the institutional protocol. Standard monitoring, including electrocardiography, non-invasive blood pressure, and pulse oximetry, was applied. Intraoperative intravenous midazolam (1-2 mg) was administered when patients exhibited clinically significant anxiety, discomfort, or restlessness.

Following surgery, patients were transferred to the postoperative recovery area once fully awake and hemodynamically stable. The primary outcome was the between-group difference in APAIS score measured 30 minutes before surgery. Secondary outcomes were between-group differences in TBR at predefined perioperative time points, the correlation between APAIS and TBR, and the intraoperative midazolam requirement.

### Statistical Analysis

Statistical analysis was performed using IBM SPSS Statistics. Normality of data distribution was assessed using the Shapiro-Wilk test. Continuous variables were expressed as medians (interquartile ranges) and compared between groups at each time point using the Mann-Whitney U test. Categorical variables were expressed as frequencies (percentages) and analyzed using the chi-square test or Fisher’s exact test, as appropriate. The correlation between APAIS scores and TBR was assessed using Spearman’s rank correlation coefficient. All tests were two-tailed; a *P *value was considered statistically significant.

## Results

**Figure 1:**
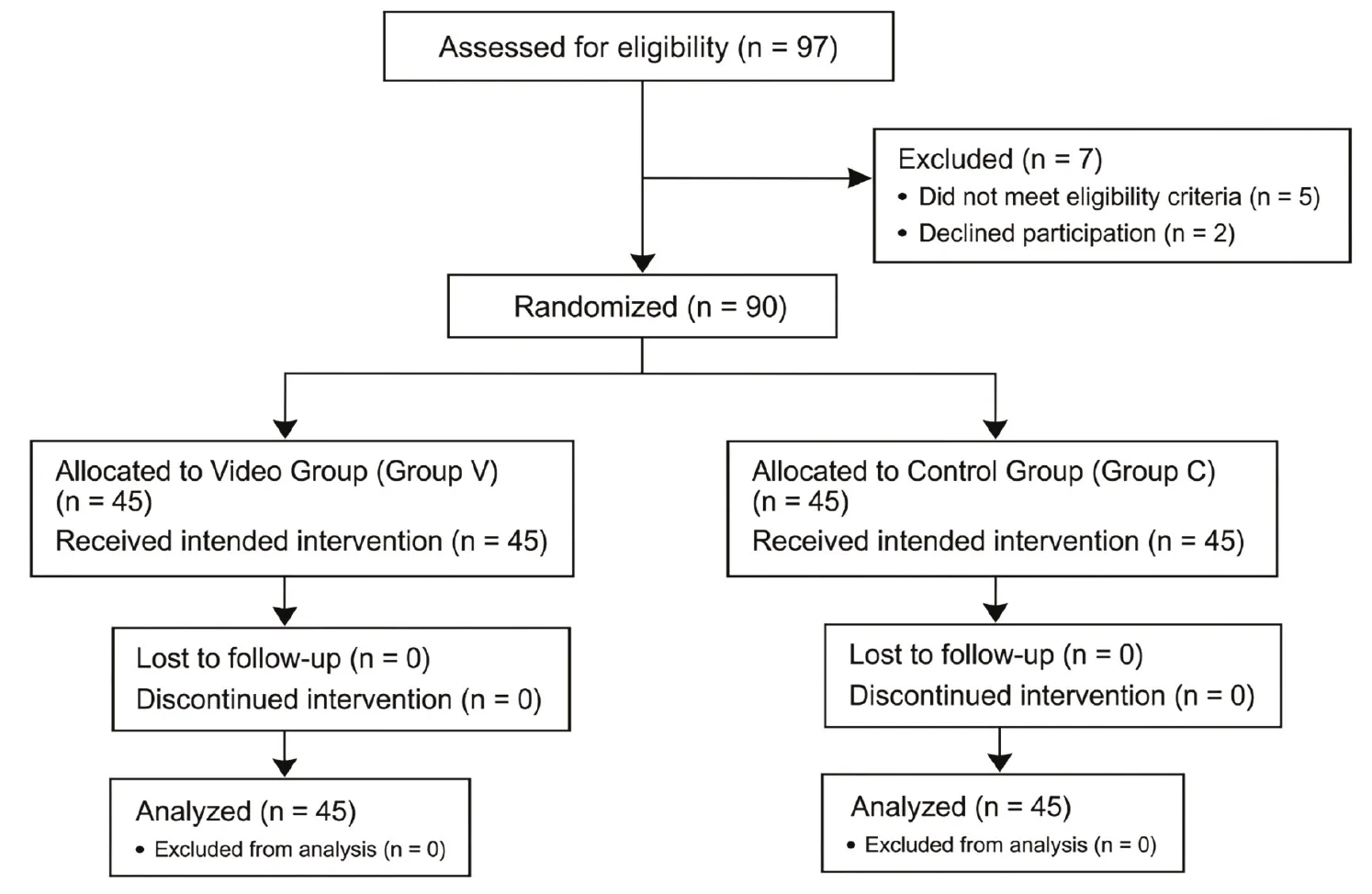
CONSORT flow diagram showing participant screening, randomization, allocation, follow-up, and analysis.

A total of 90 primigravida women undergoing elective LSCS were randomized equally into Group V (n = 45) and Group C (n = 45), with no loss to follow-up. The CONSORT flow diagram is presented in Figure 1. Baseline demographic, sociodemographic, and obstetric characteristics were comparable (Table 1). No major maternal or neonatal perioperative complications were observed in either group, and no patient required conversion to general anaesthesia.

**Figure 2:**
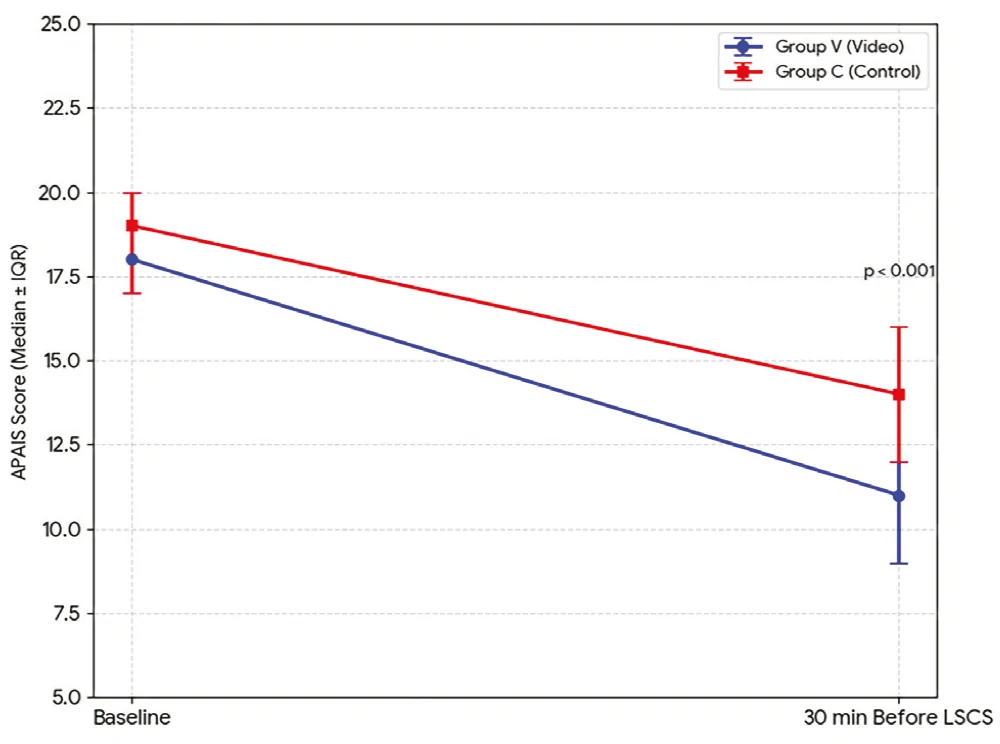
Perioperative changes in APAIS scores in the video and control groups. APAIS, Amsterdam Preoperative Anxiety and Information Scale; LSCS, lower-segment cesarean section; IQR, interquartile range.

Baseline APAIS scores were similar between the two groups, while 30 minutes before surgery, preoperative anxiety was significantly lower in Group V than in Group C (Table 2). Perioperative changes in APAIS score are illustrated in Figure 2.

**Figure 3:**
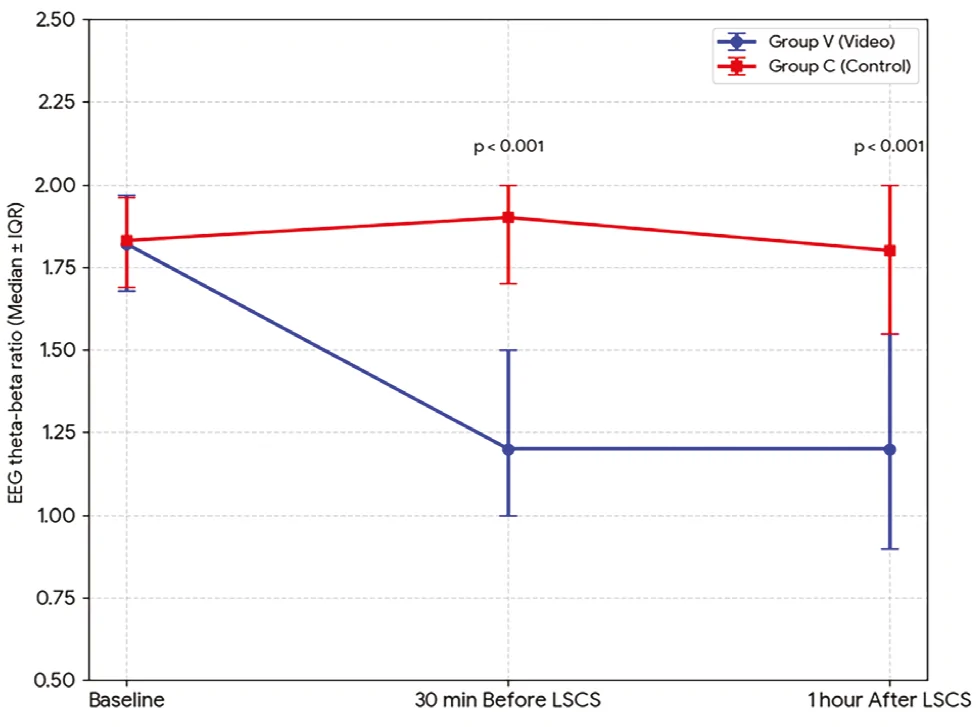
Perioperative changes in QEEG-derived TBR in the video and control groups. LSCS, lower-segment cesarean section; QEEG, quantitative electroencephalography; IQR, interquartile range; EEG, electroencephalography; TBR, theta-beta ratio.

Baseline QEEG-derived TBR was comparable between the groups. However, during the immediate preoperative period, Group V demonstrated significantly lower TBR values than Group C, and this difference persisted at 1 hour after surgery (Table 3).Perioperative changes in TBR are illustrated in Figure 3.

**Figure 4:**
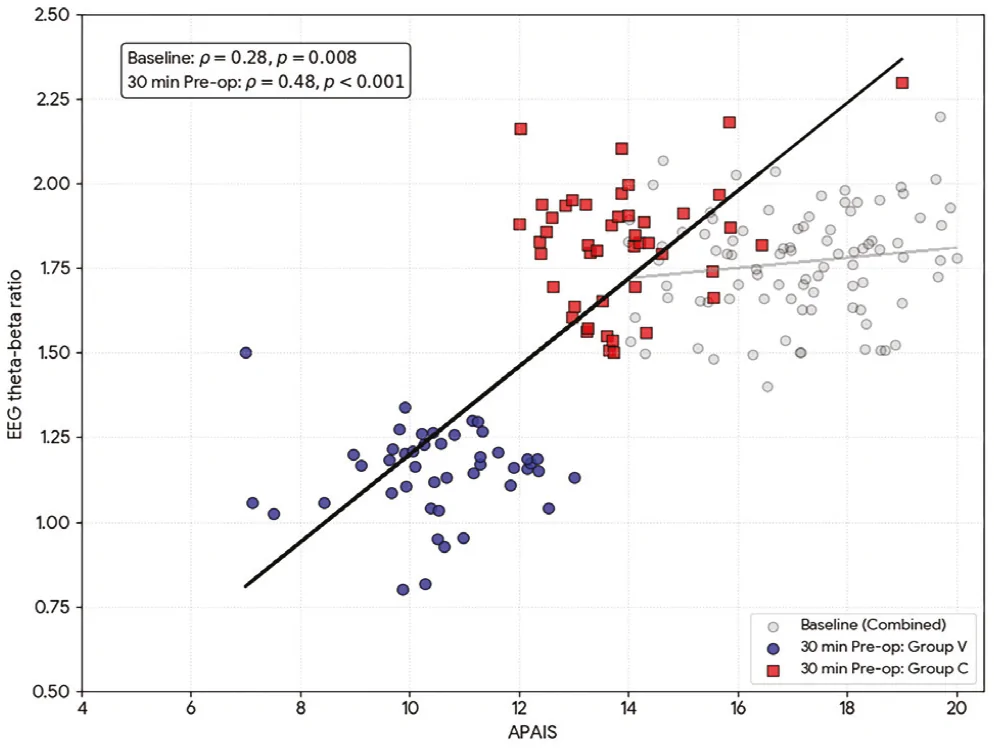
Scatter plots demonstrating the correlation between APAIS scores and QEEG-derived TBR at baseline and 30 minutes before surgery. APAIS, Amsterdam Preoperative Anxiety and Information Scale; QEEG, quantitative electroencephalography; EEG, electroencephalography; TBR, theta-beta ratio.

A positive correlation between APAIS and TBR was observed at baseline (*Ρ*=0.28, *P*=0.008) and became stronger in the immediate preoperative period (*Ρ*=0.48, *P* < 0.001), as shown in Figure 4. The requirement for intraoperative midazolam was significantly lower in the video group than in the control group (11.1% vs. 33.3%, *P*=0.021).

## Discussion

This randomized controlled trial demonstrated that a SPEV significantly reduced maternal anxiety before elective cesarean section. The intervention was associated with lower APAIS scores, lower QEEG-derived TBR values, and reduced intraoperative midazolam requirements compared with standard counseling alone. Together, these findings suggest that improved perioperative understanding may translate into both psychological and clinically relevant benefits.

Preoperative anxiety is particularly common in obstetric patients, in whom concerns regarding maternal safety, fetal well-being, anaesthesia, and the unfamiliar operating room environment may amplify distress. Previous studies have shown that heightened anxiety may be associated with increased anaesthetic and analgesic requirements, greater postoperative pain, delayed recovery, and lower patient satisfaction.^1, 2, 3, 4, 5^ Accordingly, safe and scalable non-pharmacological strategies to reduce anxiety are of considerable clinical interest.

Non-pharmacological strategies, including structured counseling, relaxation techniques, and informational videos, have increasingly been investigated to reduce perioperative anxiety by improving patient knowledge and reducing uncertainty. Several randomized and quasi-experimental studies have demonstrated that structured audiovisual education about anaesthesia and surgical procedures can reduce self-reported anxiety and improve patient satisfaction.^6, 7, 15, 16^ In the context of elective cesarean delivery, educational videos explaining anaesthesia and perioperative processes have been shown to reduce maternal anxiety and enhance perioperative satisfaction.^8, 9, 10, 11, 12^ Our findings are consistent with this body of evidence and extend previous work by incorporating an objective neurophysiological correlate of anxiety.

A notable strength of the present study is the parallel improvement observed in both subjective and objective outcomes. Women who viewed the video had lower APAIS scores 30 minutes before surgery and significantly lower TBR values during the immediate preoperative period and 1 hour after surgery. The persistence of this effect into the early postoperative period may indicate sustained attenuation of perioperative stress responses.

QEEG studies suggest that anxiety-related changes in TBR are region-specific. Frontal TBR has been associated with impaired attentional control and reduced top-down regulation, whereas temporal and parietal regions are associated with emotional arousal and internally directed attention.^13, 14^ However, findings are heterogeneous: both increased and decreased TBR have been reported, depending on underlying theta or beta activity. Temporal signals are also more susceptible to electromyographic artifacts, thereby limiting their reliability in perioperative settings. Frontal TBR, although useful for assessing executive control, may be less sensitive to anticipatory anxiety, which is characterized by rumination and expectancy.^14, 18^ In this context, parietal TBR may represent a more pragmatic marker of anticipatory anxiety in the perioperative environment.

In contrast, parietal TBR appears to be a more stable marker of internally directed attention and anticipatory processing, with evidence suggesting preferential involvement of posterior cortical networks in anxiety states.^19, 20^ In the present study, the observed reduction in parietal TBR following exposure to the educational video may reflect attenuation of anticipatory cognitive processing and emotional arousal during the immediate preoperative period. These findings support the concept that QEEG-derived biomarkers may provide complementary insights into perioperative emotional states.

The observed strengthening of the correlation between APAIS scores and TBR as surgery approached is also clinically relevant. It suggests that subjective anxiety and cortical arousal become more closely aligned during the period of greatest anticipatory stress. This supports the use of multimodal assessment strategies in perioperative anxiety research rather than relying solely on questionnaires.

The results of our study complement broader findings from research on digital and multimedia educational interventions aimed at reducing preoperative anxiety. A recent systematic review examining digital health education across multiple surgical contexts reported mixed but generally favorable effects, particularly when audiovisual information was combined with interactive elements or tailored patient education.^21^ In other surgical populations, familiarization with perioperative pathways, including explanations of nursing care, operating room procedures, and anaesthesia techniques, has been associated with reduced anxiety and stabilized physiological parameters such as heart rate and blood pressure.^22^ These findings support the premise that improving patient preparedness through structured perioperative education may enhance perioperative comfort.

From a practical perspective, this intervention is inexpensive, easy to deliver, reproducible, and readily scalable. Short educational videos can be incorporated into routine preoperative workflows using existing smartphones or tablets without exposing patients to additional medication-related adverse effects. This may be particularly valuable in resource-limited settings where staff time and pharmacological options are constrained.

Strengths of this study include its randomized controlled design, complete follow-up, and combined use of subjective and objective anxiety measures. Future multicenter studies should evaluate diverse obstetric populations, compare different video formats and digital delivery platforms, and integrate EEG with autonomic or hormonal biomarkers to better characterize perioperative anxiety. Emerging technologies such as interactive mobile applications and virtual-reality-based education may further enhance patient engagement and perioperative preparedness.^23^ Such approaches may help refine personalized, non-pharmacological strategies to improve the perioperative experience for mothers undergoing cesarean delivery.

### Study Limitation

This single-center study had a relatively modest sample size, which may limit generalizability. Participants could not be blinded to the intervention, which introduces the possibility that expectation bias influenced subjective outcomes. Additional interaction time in the intervention group may have contributed to a Hawthorne effect, which could have enhanced the observed benefit. The study population was limited to primigravida women undergoing elective cesarean section under spinal anaesthesia. Therefore, the findings may not be directly applicable to multiparous women, emergency procedures, or other surgical populations. Previous exposure to pregnancy-related online educational material or social media content was not assessed and may have influenced baseline perceptions and anxiety levels. QEEG recordings, although objective, are inherently susceptible to artifacts and environmental influences, and the use of an 8-channel system may not fully capture the complexity of neural activity associated with anxiety. Furthermore, other physiological markers of stress, such as heart rate variability and cortisol levels, were not assessed and could have provided complementary data. The study also focused on short-term perioperative outcomes, and longer-term effects on postoperative recovery, maternal satisfaction, or maternal-infant bonding were not evaluated.

Since this article is a literature review and neither of the authors included any new studies with human participants or animals, ethical approval was not required.

## Conclusion

This study suggests that a structured, validated SPEV significantly reduces preoperative anxiety in women undergoing elective LSCS, as evidenced by subjective assessments (APAIS) and objective QEEG-derived TBR biomarkers. This reduction was associated with a lower intraoperative midazolam requirement. These findings support the incorporation of low-cost audiovisual educational tools into routine preoperative care as a practical, safe, and effective non-pharmacological strategy to improve patient preparedness and reduce perioperative anxiety.

## Ethics

**Ethics Committee Approval:** The study was approved by the Institutional Ethics Committee of the All India Institute of Medical Sciences, Bhopal, New Delhi (approval no: IHEC-LOP/2023/IL0146, date: 02.01.2024).

Informed Consent: Written informed consent was obtained from all participants before enrollment.

## Figures and Tables

**Table 1. Baseline Demographic, Sociodemographic, and Obstetric Characteristics:** 

**Variable**	**Group V (n = 45)**	**Group C (n = 45)**	***P* value**
**A.** **Demographic** **characteristics**			
Age (years), mean ± SD	27.8±4.9	26.9±4.7	0.384
BMI (kg m^2-1^), mean ± SD	25.4±2.9	25.9±3.0	0.461
**B.** **Sociodemographic** **characteristics**			
Education level, n (%)			
Uneducated	4 (8.9%)	6 (13.3%)	-
High school	15 (33.3%)	18 (40.0%)	-
Graduate	18 (40.0%)	14 (31.1%)	0.721
Postgraduate	8 (17.8%)	7 (15.6%)	-
Socioeconomic status, n (%)			
Lower	12 (26.7%)	15 (33.3%)	-
Middle	24 (53.3%)	21 (46.7%)	0.684
Upper-middle	9 (20.0%)	9 (20.0%)	-
Occupation, n (%)			
Housewife	30 (66.7%)	27 (60.0%)	-
Government employee	8 (17.8%)	10 (22.2%)	0.793
Private sector employee	7 (15.6%)	8 (17.8%)	-
Residence, n (%)			
Rural	24 (53.3%)	27 (60.0%)	0.672
Urban	21 (46.7%)	18 (40.0%)	-
**C.** **Obstetric** **characteristics**			
Gestational age (weeks), mean ± SD	38.5±1.9	38.7±1.5	0.557
Previous hospitalization, n (%)	11 (24.4%)	13 (28.9%)	0.814

BMI, body mass index; SD, standard deviation

**Table 2. Comparison of APAIS Scores Between Groups at Different Time Points:** 

**Time points**	**Group V(n = 45)**	**Group C(n = 45)**	***P* value**
Baseline	18.0 (17.0-20.0)	19.0 (17.0-20.0)	0.85
30 min before LSCS	11.0 (9.0-12.0)	14.0 (12.0-16.0)	<0.001

LSCS, lower-segment cesarean section; APAIS, Amsterdam Preoperative Anxiety and Information Scale

**Table 3. Comparison of QEEG Theta-Beta Ratio Between Groups at Different Time Points:** 

**Time points**	**Group V(n = 45)**	**Group C(n = 45)**	***P*** **value**
Baseline	1.82 (1.68-1.97)	1.83 (1.69-1.96)	0.94
30 min before LSCS	1.20 (1.00-1.50)	1.90 (1.70-2.00)	<0.001
1 hour after LSCS	1.20 (0.90-1.55)	1.80 (1.55-2.00)	<0.001

LSCS, lower-segment cesarean section; QEEG, quantitative electroencephalography
